# Polarization based discrete variables quantum key distribution via conjugated homodyne detection

**DOI:** 10.1038/s41598-022-10181-4

**Published:** 2022-04-12

**Authors:** Mariana F. Ramos, Armando N. Pinto, Nuno A. Silva

**Affiliations:** 1 Instituto de Telecomunicações, University of Aveiro, Campus Universitário de Santiago, 3810-193 Aveiro, Portugal; 2 Department of Electronics, Telecommunications and Informatics, University of Aveiro, Campus Universitário de Santiago, 3810-193 Aveiro, Portugal

**Keywords:** Optics and photonics, Physics

## Abstract

Optical homodyne detection is widely adopted in continuous-variable quantum key distribution for high-rate field measurement quadratures. Besides that, those detection schemes have been being implemented for single-photon statistics characterization in the field of quantum tomography. In this work, we propose a discrete-variable quantum key distribution (DV-QKD) implementation that combines the use of phase modulators for high-speed state of polarization (SOP) generation, with a conjugate homodyne detection scheme which enables the deployment of high speed QKD systems. The channel discretization relies on the application of a detection threshold that allows to map the measured voltages as a click or no-click. Our scheme relies also on the use of a time-multiplexed pilot tone—quantum signal architecture which enables the use of a Bob locally generated local oscillator and opens the door to an effective polarization drift compensation scheme. Besides that, our results shows that for higher detection threshold values we obtain a very low quantum bit error rate (QBER) on the sifted key. Nevertheless, due to huge number of discarded qubits the obtained secure key length abruptly decreases. From our results, we observe that optimizing the detection threshold and considering a system operating at 500 MHz symbol generation clock, a secure key rate of approximately 46.9 Mbps, with a sifted QBER of  $1.5\%$ over 40 km of optical fiber. This considering the error correction and privacy amplification steps necessary to obtain a final secure key.

## Introduction

Currently, the digital data that evolves in the telecommunication networks is secured based on classical protocols that rely on computational complexity^1^. However, with both the rapid development of supercomputers and the imminent emergence of a practical quantum computer, most of those asymmetric cryptography protocols may rapidly become insecure^2^. In contrast with computational complexity based security techniques, security based on the physical-layer properties leads to robust communication systems even against an eavesdropper with unlimited computational power. Quantum key distribution (QKD) is one of those systems, where security relies on quantum physics laws, which assures secret correlations unconditionally secure between parties assuming a certain level of trust on the used devices^3,4^.

QKD protocols can be implemented following two fundamental approaches. In DV-QKD, information is encoded in one (or more) degree-of-freedom of individual photons, which leads to a discrete measurement outcome^5^. Assuring compatibility with current telecommunication infrastructures, CV-QKD schemes use multi-photon quantum states of light encoding the bits using observables with the continuous variables such as the phase and amplitude of coherent states^6^. DV-QKD schemes have been experimentally demonstrated over long distances^7,8^, and present more mature security proofs taking into account system imperfections and finite data size effects^9^. On the other hand, CV-QKD schemes allow to achieve higher transmission rates at short distances on current telecommunication metro networks^10^. Remarkable technological advances have been done in QKD systems aiming to improve the transmission rate, achievable distance and decrease the implementation cost^11^. High-speed measurement-device-independent QKD systems have been experimental demonstrated using GHz clock rates, where 2 kbps secrete key rates were obtained in a finite-size regime and over 180 km channel length^12,13^. Moreover, the implementation cost of such systems has been also being reduced^14^, and long-distance QKD demonstrations folowwing towards of quantum secure networks over a 1000 km scale^15–17^.

Despite some disadvantages of CV-QKD arise mainly from the complexity of information reconciliation steps^6^, their compatibility with classical detection hardware poses a major advantage against current single-photon avalanche based detection schemes required for the DV-QKD, which limits on the achievable performance and work at very-low temperatures demanding additional cooling systems^18^. Discrete-modulated CV-QKD were also proposed over 100 km optical fibers, where a discrete modulation of quantum states is used in conjunction with homodyne detection schemes^19,20^. More recently, a detection scheme to determine the photon number statistics of an input quantum state using conjugate homodyne detection without controlling the phase of the input quantum state was proposed^21^. The photon number statistics is one of the research tasks on quantum tomography^22^, where homodyne detection has been being implemented for that purpose^23^. Later, a DV-QKD implementation was presented using a conjugate homodyne detection scheme that operates in counting mode. This detection scheme consists on a polarization beam splitter (PBS) followed by two optical homodyne detectors, which allows the measurement of a pair of quadratures of the input quantum state^24^. Although most of the homodyne detection schemes used to decode single-photons assume ideal single-photon sources, an hybrid solution based on decoy-state and homodyne detection was proposed in^25^, where the local oscillator phase is randomised being no need to distribute a common phase reference between transmitter and receiver. Due to the non-practical conditions required to create ideal single-photon sources, experimental DV-QKD is implemented using coherent state sources highly attenuated to an average number of 0.1 photons per pulse^26^. DV-QKD systems considering non-ideal single-photon sources was experimental demonstrated, which are tolerant to channel losses even considering source imperfections to generate non-ideal quantum states^27,28^. Furthermore, other experimental demonstrations was presented considering another system imperfections, for instance optical devices and post-processing classical units possibly controlled by an eavesdropper^29^ and the existence of polarization-dispersion loss over silicon-based phase modulators^30^. Moreover, the switching between states of polarization (SOP) using phase modulators allows SOP generation rates in the order of GHz^31^. Current state-of-the-art reports a BB84 quantum states generation at 5 GHz pulse repetition rate over 151.5 km using a phase modulator to encode quantum information on single-photons polarization, achieving a final secret key rate of 54.5 kbps^31^. This kind of technique provides optical pulse modulation within the acceptance bandwidth of the phase modulators with high extinction ratio^32^.

In this work, we propose a novel polarization-based DV-QKD system that combines the use of phase-modulators to SOP generation and basis switching with a polarization diversity coherent detection scheme. This enables a full implementation of DV-QKD systems using only classical hardware. At transmitter side, high-baud rate low-intensity quantum signals are enabled by using a highly attenuated laser source, and a Mach–Zehnder Modulator followed by 45$^{\circ }$ aligned Phase Modulator. At receiver side, random basis choice by Bob can be performed using also a 45$^{\circ }$ aligned Phase Modulator followed by a commercial integrated polarization-diversity coherent receiver. We also propose the implementation of quantum frames with time-multiplexing pilot tone sent by the transmitter to enable the use of a locally generated oscillator at receiver. Our results open the door to polarization qubits transmission baud-rates of the order of GHz in access and metro networks. We report continuous qubit transmission, even in environments subjected to high polarization drift, without consuming extra-bandwidth with a maximum $2\%$ QBER. Furthermore, we report a secure key generation rate of approximately 46.9 Mbps, with a sifted QBER of  $1.5\%$, and a detection threshold of 0.87 mV, when implementing the BB84 protocol in a system operating at a 1 GHz symbol generation clock over 40 km of standard optical fibers.

This paper contains four sections. First, we detail the theoretical model of the proposed polarization based DV-QKD system. Next, we detail the DV-QKD BB84 protocol implementation in the proposed system, assess the method for polarization compensation, and we also assess the performance of BB84 protocol in a finite-key size implementation using thresholds to operate the proposed system in counting mode. Finally, in the last section, the main conclusion of the presented work are summarized.

## DV-QKD polarization diversity coherent detection based system

**Figure 1:**
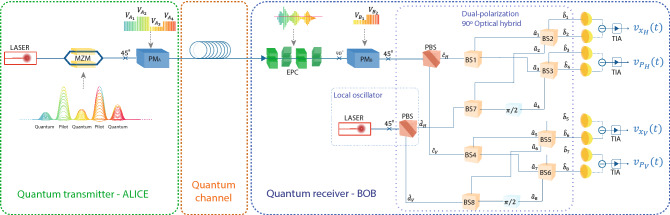
Schematic representation of the discrete variable quantum key distribution (DV-QKD) system based on polarization diversity coherent detection. [MZM] denotes the Mach–Zehender amplitude modulator, [$\text {PM}_\text {A}$] and [$\text {PM}_\text {B}$] the phase-modulators of Alice and Bob, respectively, [EPC] the electronic polarization controller, [PBS] the polarization beam-splitters, [BS] the beam-splitters, and [TIA] the trans-impedance amplifiers.

In this section, we present the theoretical model of the proposed polarization based DV-QKD system that combines the usage of phase modulators to generate quantum polarized states with a polarization diversity coherent detection scheme. The transmitter, usually known as Alice, randomly generates the BB84 states using phase-randomised weak coherent pulses, and the receiver, usually known as Bob, performs random quadrature measurements. Figure 1 shows the schematic representation of the proposed polarization based DV-QKD transmission system, which is divided in three parts namely Alice, the quantum channel, and Bob.

### Polarization state preparation

Alice generates the BB84 polarization states by combining a weak coherent optical signal source, as an approximation to a true single-photon source, with a phase-modulator to switch between the four possible states of polarization. In order to guarantee the security under current security analyses, the proposed system implements phase randomization^33,34^, which by exploiting the non-orthogonal coding allows the use of two or more photon component to obtain the secrete key^35–39^. In order to increase security avoiding for instance photon number splitting attacks to this photon source, the security could be increased significantly if we also implement a decoy-state protocol. Please note that, in literature it was already proved that the use of a weak-coherent optical signal in the DV-QKD BB84 protocol implemented together with a decoy-state protocol leads to an unconditional secure QKD implementation^40,41^. The polarization state preparation scheme consists on a single-laser source followed by a Mach–Zehender (MZM) amplitude modulator, and a phase modulator ([$\text {PM}_\text {A}$)^42^. Alice applies time-division multiplexing techniques to transmit pulses with different amplitudes by switching between two voltage levels on consecutive pulses of the signal that drives the MZM, see Fig. 1. One of those levels correspond to the high power pilot tone, which is sent to enable the use of a locally generated local oscillator and to reverse the polarization random drift that the photons suffers during its evolution over the quantum channel. The other voltage level corresponds to the weak coherent optical signal in such a way to obtain 0.2 photons per pulse on average, which corresponds to the information carried by the quantum state. At the MZM output the annihilation operator is a well defined horizontal polarized optical pulse that can be defined as^43^,

1 $$\begin{aligned} {\hat{a}}_{\text {in}_H}(t)= \sqrt{\eta _{\text {MZM}}(t-nT_s)}{\hat{a}}_{0_H} e^{i(\omega _st+\phi _{s_N}(t))} h(t-nT_s), \end{aligned}$$

where $\eta _{MZM}(t-nT_s)$ is the MZM efficiency over the symbol duration ($T_s$) of each pulse with symbol number *n*, ${\hat{a}}_{0_H}$ denotes the annihilation quantum operator of a coherent state of a single-mode laser^44^, $\omega _s$ is the optical frequency of the quantum signal, $\phi _{s_N}(t)$ is the initial unknown optical phase of the laser, and $h(t-nT_s)$ denotes the pulse shape signal of MZM^45^. Please note that we consider that the polarization state at the laser optical signal output is a well defined horizontal polarization state. In this work we consider a return-to-zero pulse with $50\%$ duty cycle. From Eq. (1), we can define the average number of photons per quantum pulse, $\langle n_{Q}\rangle = \langle \alpha _L| {\hat{a}}^{\dagger }_{\text {in}_H}(t) {\hat{a}}_{\text {in}_H}(t) |\alpha _L \rangle$ being $|\alpha _L \rangle$ the coherent state describing the laser field^44^, given by

2 $$\begin{aligned} \langle n_{Q}\rangle = \left| \alpha _s\right| ^2 \eta _{\text {MZM}}(t-nT_s)\int _{\frac{T_s}{2}(2n-1)}^{\frac{T_s}{2}(2n+1)} dt \left| h(t-nT_s)\right| ^2, \end{aligned}$$

where, $|\alpha _s|^2=P_s/(\hbar \omega _s)$ is the time-independent optical photon flux at laser output, being $P_s$ the optical power at laser output and $\hbar$ the reduced Plank constant. Note that for $\eta _{\text {MZM}}(t-nT_s) =1$ implies that we are generating a pilot tone. However, for $\eta _{\text {MZM}}(t-nT_s) \ll 1$ we are operating in a quantum regime, and in that case we are generating the quantum signals for QKD implementation.

Following the MZM in Fig.  1, the phase modulator $\text {PM}_A$ is responsible for polarization modulation. The input of the phase modulator has a polarization maintaining optical fiber oriented at $45^\circ$ with respect to the optical axis, which results in the two orthogonal equal amplitude polarization components of the electromagnetic field that’s propagate in the crystal experiencing different refractive indexes^46^. We can switch between four states of polarization by applying four different voltages at phase modulator, in particular 0, $V_\pi$, $V_{\pi /2}$, and $-V_{\pi /2}$ to obtain $|45\rangle$, $|-45\rangle$, $|RC\rangle$, and $|LC\rangle$, respectively^46^. In this work, we assume the existence of polarization dependent loss (PDL) effect in the phase modulator, which is defined as the ratio between the maximum over the minimum optical transmission coefficient, $\epsilon _{\text {PDL}}$. The maximum transmission is assumed to be 1. In this way, $\epsilon _{\text {PDL}}$ corresponds to the minimum transmission, with $\text {PDL}_{dB}= 10 \log {1/\varepsilon _{\text {PDL}}}$^47^. The two orthogonal amplitude polarization components of the electromagnetic field at Alice output can be defined in terms of quantum annihilation operators as^43,44,48^,

3 $$\begin{aligned} {\left\{ \begin{array}{ll} {\hat{a}}_{A_H}(t) = \frac{1}{\sqrt{2}}e^{i\frac{V_A(t-nT_s)}{V_\pi }\pi }\left( {\hat{a}}_{\text {in}_H}(t)-{\hat{a}}_{\text {in}_V}(t)\right) \\ {\hat{a}}_{A_V}(t) = \frac{1}{\sqrt{2}}\left( {\hat{a}}_{\text {in}_H}(t)-{\hat{a}}_{\text {in}_V}(t)\right) \sqrt{\varepsilon _{\text {PDL}}}, \end{array}\right. } \end{aligned}$$

where $V_A(t-nT_s)$ is the voltage applied on the phase-modulator $\text {PM}_A$ to generate one of the four BB84 polarization states, $V_\pi$ is the voltage needed to apply a phase difference of $\pi$ on the phase-modulator, and ${\hat{a}}_{in_V}$ corresponds to annihilation operator for the vertical polarization state at phase modulator output, which is in a vacuum state since the laser is assumed to emit photons only over the horizontal polarization state.

### Transmission of the polarization states over an optical channel

The quantum channel is assumed to be a standard optical fiber. We consider the polarization mode dispersion (PMD) following the work presented in^49^. The PMD degrades the transmitted state of polarization inducing random drift polarization due the birefringence inherent of the standard optical fiber channel. Polarization states change accordingly with a random matrix parameterized by the random parameters $\gamma _n = (\gamma _1,\gamma _2,\gamma _3)$ generated at each instant, where $\gamma _n = \psi \mathbf{a }$, with length $\psi = \Vert \gamma _n\Vert$, denoting $\Vert \cdot \Vert$ the euclidean norm. The randomness of rotations is defined by $\gamma _n$ parameters obtained from a normal distribution with mean zero and standard deviation $\sigma ^2 = 2 \pi \Delta _p T$, being *T* the total acquisition time and $\Delta _p$ the polarization linewidth that defines the the random drift velocity^49^. Therefore, the temporal drift evolution is modelled by concatenating consecutive matrices,

4 $$\begin{aligned} \text {M}_{\text {F}}(\gamma _n) = \mathbf{I } \cos {\psi } - i \mathbf{a } \cdot \vec {\sigma }\sin {\psi }, \end{aligned}$$

where $\vec {\sigma }$ is the tensor of Pauli matrices, $\mathbf{I }$ is a $2\times 2$ identity matrix^49^, and $\mathbf{a } = (a_1,a_2,a_3)$ denotes the direction defined in a unitary sphere. We also consider the optical fiber channel losses, which are modelled using the beam-splitter model. The transitivity of the channel is defined as $\tau _{ch}=10^{-\alpha _L L_f/10}$, where $\alpha _L$ is the dB/km attenuation coefficient, and $L_f$ the channel length in km.

### Polarization states measurement

The states of polarization enter on Bob’s measurement setup and pass through an electronic polarization controller (EPC), that is used to compensate the polarization random drift suffered over the transmission channel. In order to compensate the polarization PDL from both phase modulators, we apply a $90^\circ$ rotation to the light field before entering in $\text {PM}_B$ in Fig. 1. The component that passes through the ordinary axis in $\text {PM}_A$ crystal follows the extraordinary axis in $\text {PM}_B$, and vice-versa^46^. The phase modulator output optical fiber is spliced at $45^\circ$ applying an inverse rotation of the one performed at $\text {PM}_A$ input allowing Bob to decipher the received information correctly^46^. In this work, we assume equal phase modulators in Alice and Bob considering the same characteristics including the same PDL in both. Moreover, Bob must apply two voltage levels on the phase modulator to choose the measurement basis for turning the states into horizontal and vertical. For instance, $V_{B_1}=0$ V is applied to measure in the diagonal basis, and $V_{B_2}=V_{\pi /2}$ V to measure in the circular basis. After passing through Bob phase modulator $\text {PM}_B$, the annihilation operators for horizontal polarization state can be written as^43,48,50^,

5 $$\begin{aligned} {\hat{a}}_{B_H}(t)= & {} -\sqrt{\frac{\tau _{ch}}{2}} \left( {\mathbf {Z}}_{21}(t-nT_s)e^{i\frac{V_B(t-nT_s)}{V_\pi }\pi }+{\mathbf {Z}}_{11}(t-nT_s)\sqrt{\varepsilon _{\text {PDL}}}\right) {\hat{a}}_{A_H}(t) \nonumber \\&\quad -\sqrt{\frac{\tau _{ch}}{2}}\left( {\mathbf {Z}}_{22}(t-nT_s)e^{i\frac{V_B(t-nT_s)}{V_\pi }\pi }+{\mathbf {Z}}_{12}(t-nT_s)\sqrt{\varepsilon _{\text {PDL}}}\right) {\hat{a}}_{A_V}(t)\nonumber \\&\quad - (\text {terms associated with vacuum operators}). \end{aligned}$$

On the other hand, for the vertical polarization state, the annihilation operator can be written as^43,48,50^,

6 $$\begin{aligned} {\hat{a}}_{B_V}(t)= & {} -\sqrt{\frac{\tau _{ch}}{2}} \left( {\mathbf {Z}}_{21}(t-nT_s)e^{i\frac{V_B(t-nT_s)}{V_\pi }\pi }-{\mathbf {Z}}_{11}(t-nT_s)\sqrt{\varepsilon _{\text {PDL}}}\right) {\hat{a}}_{A_H}(t) \nonumber \\&\quad -\sqrt{\frac{\tau _{ch}}{2}}\left( {\mathbf {Z}}_{22}(t-nT_s)e^{i\frac{V_B(t-nT_s)}{V_\pi }\pi }-{\mathbf {Z}}_{12}(t-nT_s)\sqrt{\varepsilon _{\text {PDL}}}\right) {\hat{a}}_{A_V}(t)\nonumber \\&\quad - (\text {terms associated with vacuum operators}). \end{aligned}$$

In Eq. (5) and in Eq. (6) ${\mathbf {Z}}$ is the concatenation of the EPC matrix with $M_F$, see Eq. (4), $\tau _{ch}$ is the transmissivity of the optical fiber which accounts for the fiber loss, and $V_B$ is the voltage applied on $\text {PM}_B$ for changing the measurement basis. The terms associated with the vacuum operator are hidden, since they do not contribute for the average value neither for variance calculations. At the input of the dual-polarization optical hybrid in Fig. 1, the quantum signal is mixed with a strong local oscillator for quadrature measurement. The quantum operator for this second laser source that generates the local oscillator can be defined as

7 $$\begin{aligned} {\hat{a}}_{\text {Lo}_H}(t) = \left| \bar{a}_{\text {lo}_H}\right| e^{i(\omega _{lo}t+\phi _{lo_N}(t))}, \end{aligned}$$

where $\bar{a}_{lo_H}$ is the (classical) amplitude of the local oscillator laser, $\omega _{Lo}$ is the optical frequency of the local oscillator, and $\phi _{Lo}$ is the optical phase of the local oscillator.

#### Voltages at Bob homodyne detection outputs

After being detected by each pair of photo-diodes, the electrical signals are subtracted and amplified by a trans-impedance amplifier (TIA) following a standard homodyne detection scheme. The four voltages after the TIA obtained at the Bob homodyne detection scheme output in Fig. 1 for a given symbol *n* are given by^43,44,51,52^,

8 $$\begin{aligned} v_{q_p}^{(n)}(t)=g_{\text {TIA}} \int _{-\infty }^{t}d\tau '\big \langle {\hat{i}}_{q_p}(t-\tau ') \big \rangle r_{\text {TIA}}(\tau '), \end{aligned}$$

where $q= \{ X, P\}$ denotes the quadrature, and $p=\{H,V\}$ denotes the corresponding polarization, and ${\hat{i}}_{q_p}(t)$ represents the current generated by the homodyne detector^51^. Moreover, in Eq. (8) the $g_{\text {TIA}}$ is the TIA’s gain, and $r_{\text {TIA}}(t)$ denotes the Fourier transform of the impulse response function considering a Butterworth filter of order *m* and bandwidth $B_e$ given in frequency domain by^51^

9 $$\begin{aligned} H(\omega ) = \frac{1}{\left[ 1+ \left( \frac{\omega }{2\pi B_e}\right) ^{2 m}\right] ^{1/2}}, \end{aligned}$$

where $B_e$ is the filter bandwidth. In this work we assume a Butterworth filter with $m=1$, ideal digital signal processing for phase and frequency carrier recovery. Besides that, we also assume that the parameters ${\mathbf {Z}}_{ij}(t-nT_s)$, $\eta _{\text {MZM}}(t-nT_s)$, $V_A(t-nT_s)$, and $V_B(t-nT_s)$ are constant within a given pulse *n*, they only can change between optical pulses. In that sense those parameters can be written as ${\mathbf {Z}}_{ij}(t-nT_s) \approx {\mathbf {Z}}^{(n)}_{ij}$, $\eta _{\text {MZM}}(t-nT_s) \approx \eta _{\text {MZM}}^{(n)}$, $V_A(t-nT_s) \approx V_A^{(n)}$, and $V_B(t-nT_s) \approx V_B^{(n)}$. The expected value of the current at the output of the each homodyne detector (quantum signal or pilot tone) in Fig. 1 and for each transmitted symbol *n* is given by,

10a $$\begin{aligned} \big \langle {\hat{i}}_{x_H}(t) \big \rangle= & {} q_e \big \langle {\hat{a}}_1^\dagger (t){\hat{a}}_2(t) + {\hat{a}}_2^\dagger (t){\hat{a}}_1(t) \big \rangle = -\frac{1}{2\sqrt{2}}q_e\sqrt{\eta _d}\sqrt{\tau _{ch}} \sqrt{\eta _{\text {MZM}}^{(n)}} \left| \alpha _{Lo}\right| \left| \alpha _s \right| \nonumber \\&\quad \times \text {Re} \left\{ \left( {\mathbf {Z}}_{21}^{(n)}e^{i\frac{V_B^{(n)}}{V_\pi }\pi }+ {\mathbf {Z}}_{11}^{(n)}\sqrt{\varepsilon _{\text {PDL}}}\right) e^{i\frac{V_A^{(n)}}{V_\pi }\pi }+\left( {\mathbf {Z}}_{22}^{(n)}e^{i\frac{V_B^{(n)}}{V_\pi }\pi }+{\mathbf {Z}}_{12}^{(n)}\sqrt{\varepsilon _{\text {PDL}}} \right) \sqrt{\varepsilon _{\text {PDL}}}\right\} h(t-nT_s), \end{aligned}$$

10b $$\begin{aligned} \big \langle {\hat{i}}_{p_H}(t) \big \rangle= & {} q_e \big \langle {\hat{a}}_3^\dagger (t){\hat{a}}_4(t) + {\hat{a}}_4^\dagger (t){\hat{a}}_3(t) \big \rangle = -\frac{1}{2\sqrt{2}}q_e\sqrt{\eta _d}\sqrt{\tau _{ch}} \sqrt{\eta _{\text {MZM}}^{(n)}} \left| \alpha _{Lo}\right| \left| \alpha _s \right| \nonumber \\&\quad \times \text {Im} \left\{ \left( {\mathbf {Z}}_{21}^{(n)}e^{i\frac{V_B^{(n)}}{V_\pi }\pi }+ {\mathbf {Z}}_{11}^{(n)}\sqrt{\varepsilon _{\text {PDL}}}\right) e^{i\frac{V_A^{(n)}}{V_\pi }\pi }+ \left( {\mathbf {Z}}_{22}^{(n)}e^{i\frac{V_B^{(n)}}{V_\pi }\pi }+{\mathbf {Z}}_{12}^{(n)}\sqrt{\varepsilon _{\text {PDL}}} \right) \sqrt{\varepsilon _{\text {PDL}}}\right\} h(t-nT_s), \end{aligned}$$

10c $$\begin{aligned} \big \langle {\hat{i}}_{x_V}(t) \big \rangle= & {} q_e \big \langle {\hat{a}}_5^\dagger (t){\hat{a}}_6(t) + {\hat{a}}_6^\dagger (t){\hat{a}}_5(t) \big \rangle = -\frac{1}{2\sqrt{2}}q_e\sqrt{\eta _d}\sqrt{\tau _{ch}} \sqrt{\eta _{\text {MZM}}^{(n)}} \left| \alpha _{Lo}\right| \left| \alpha _s \right| \nonumber \\&\quad \times \text {Re} \left\{ \left( {\mathbf {Z}}_{21}^{(n)}e^{i\frac{V_B^{(n)}}{V_\pi }\pi }- {\mathbf {Z}}_{11}^{(n)}\sqrt{\varepsilon _{\text {PDL}}}\right) e^{i\frac{V_A^{(n)}}{V_\pi }\pi }+ \left( {\mathbf {Z}}_{22}^{(n)}e^{i\frac{V_B^{(n)}}{V_\pi }\pi }-{\mathbf {Z}}_{12}^{(n)}\sqrt{\varepsilon _{\text {PDL}}} \right) \sqrt{\varepsilon _{\text {PDL}}}\right\} h(t-nT_s), \end{aligned}$$

10d $$\begin{aligned} \big \langle {\hat{i}}_{p_V}(t) \big \rangle= & {} q_e \big \langle {\hat{a}}_7^\dagger (t){\hat{a}}_8(t) + {\hat{a}}_8^\dagger (t){\hat{a}}_6(t) \big \rangle = -\frac{1}{2\sqrt{2}}q_e\sqrt{\eta _d}\sqrt{\tau _{ch}} \sqrt{\eta _{\text {MZM}}^{(n)}} \left| \alpha _{Lo}\right| \left| \alpha _s \right| \nonumber \\&\quad \times \text {Im} \left\{ \left( {\mathbf {Z}}_{21}^{(n)}e^{i\frac{V_B^{(n)}}{V_\pi }\pi }- {\mathbf {Z}}_{11}^{(n)}\sqrt{\varepsilon _{\text {PDL}}}\right) e^{i\frac{V_A^{(n)}}{V_\pi }\pi }+ \left( {\mathbf {Z}}_{22}^{(n)}e^{i\frac{V_B^{(n)}}{V_\pi }\pi }-{\mathbf {Z}}_{12}^{(n)}\sqrt{\varepsilon _{\text {PDL}}} \right) \sqrt{\varepsilon _{\text {PDL}}}\right\} h(t-nT_s), \end{aligned}$$

where $\eta _D$ denotes the detection efficiency, $q_e$ is the charge of the electron, and $\big |\alpha _{\text {Lo}}\big |^2$ is the optical flux of the locally generated local oscillator. Note that ${\hat{a}}_i^\dagger (t) {\hat{a}}_j(t)$, with $i,j = 1,2,3,4,5,6,7,8$, is the optical flux in each branch of the BS output in Fig. 1. Accordingly with the expected value of the currents defined in Eq. (10), and the voltage-current relation defined in Eq. (8), the measured quadratures for a given transmitted symbol *n* are defined by integrating the homodyne voltage over a certain time interval^43,44,51,52^,

11a $$\begin{aligned} {\hat{Q}}_{H,n}= & {} \frac{1}{T_s} \int _{\frac{T_s}{2}(2n-1)}^{\frac{T_s}{2}(2n+1)} v_{x_H}^{(n)}(t) = \frac{1}{T_s}g_{\text {TIA}}\int _{\frac{T_s}{2}(2n-1)}^{\frac{T_s}{2}(2n+1)}dt \int _{-\infty }^{t}d\tau ' \big \langle {\hat{i}}_{x_H}(t-\tau ') \big \rangle r_{\text {TIA}}(\tau ') + {\hat{Q}}_{e,n} + {\hat{Q}}_{S_{X,H},n}, \end{aligned}$$

11b $$\begin{aligned} {\hat{P}}_{H,n}= & {} \frac{1}{T_s} \int _{\frac{T_s}{2}(2n-1)}^{\frac{T_s}{2}(2n+1)} v_{p_H}^{(n)}(t) = \frac{1}{T_s}g_{\text {TIA}}\int _{\frac{T_s}{2}(2n-1)}^{\frac{T_s}{2}(2n+1)}dt \int _{-\infty }^{t}d\tau ' \big \langle {\hat{i}}_{p_H}(t-\tau ') \big \rangle r_{\text {TIA}}(\tau ') + {\hat{Q}}_{e,n} + {\hat{Q}}_{S_{P,H},n}, \end{aligned}$$

11c $$\begin{aligned} {\hat{Q}}_{V,n}= & {} \frac{1}{T_s} \int _{\frac{T_s}{2}(2n-1)}^{\frac{T_s}{2}(2n+1)} v_{x_V}^{(n)}(t) = \frac{1}{T_s}g_{\text {TIA}}\int _{\frac{T_s}{2}(2n-1)}^{\frac{T_s}{2}(2n+1)}dt \int _{-\infty }^{t}d\tau ' \big \langle {\hat{i}}_{x_V}(t-\tau ') \big \rangle r_{\text {TIA}}(\tau ') + {\hat{Q}}_{e,n} + {\hat{Q}}_{S_{X,V},n}, \end{aligned}$$

11d $$\begin{aligned} {\hat{P}}_{V,n}= & {} \frac{1}{T_s} \int _{\frac{T_s}{2}(2n-1)}^{\frac{T_s}{2}(2n+1)} v_{p_V}^{(n)}(t) = \frac{1}{T_s}g_{\text {TIA}}\int _{\frac{T_s}{2}(2n-1)}^{\frac{T_s}{2}(2n+1)}dt \int _{-\infty }^{t}d\tau ' \big \langle {\hat{i}}_{p_V}(t-\tau ') \big \rangle r_{\text {TIA}}(\tau ') + {\hat{Q}}_{e,n} + {\hat{Q}}_{S_{P,V},n}, \end{aligned}$$

where ${\hat{Q}}_{e,n}$ is the electronic noise due to the TIA for each transmitted symbol *n*, and ${\hat{Q}}_{S_{q,p},n}$ is the shot noise. The variance of the quadratures in Eq. (11) for a given optical transmitted pulse *n* is given by^43,44,51,52^,

12 $$\begin{aligned} \sigma _{q_p}^2= & {} \frac{g_{\text {TIA}}^2}{T_s^2}\int _{\frac{T_s}{2}(2n-1)}^{\frac{T_s}{2}(2n+1)} dt_1\int _{\frac{T_s}{2}(2n-1)}^{\frac{T_s}{2}(2n+1)} dt_2 \int _{-\infty }^{t_1}d\tau ' \int _{-\infty }^{t_2}d\tau '' \left[ \big \langle {\hat{i}}_{q_p}(t_1-\tau '){\hat{i}}_{q_p}(t_2-\tau '') \big \rangle \right. \nonumber \\&\quad - \left. \big \langle {\hat{i}}_{q_p}(t_1-\tau ') \big \rangle \big \langle {\hat{i}}_{q_p}(t_2-\tau '') \rangle \right] r_{\text {TIA}}(\tau ')r_{\text {TIA}}(\tau '')+ Q_{e,n}^2. \end{aligned}$$

In Eq. (12) for each quadrature $q= \{ X, P\}$ the second moment operator for the currents at homodyne detector can be written as,

13 $$\begin{aligned} \big \langle {\hat{i}}_{q_p}(t'){\hat{i}}_{q_p}(t') \big \rangle -\big \langle {\hat{i}}_{q_ p}(t') \big \rangle \big \langle {\hat{i}}_{q_p}(t') \big \rangle = \frac{1}{8}q_e^2\eta _d \left( \big \langle {\hat{a}}_{B_p}^\dagger (t'){\hat{a}}_{B_p}(t')\big \rangle +\big \langle {\hat{a}}_{Lo_H}^\dagger (t'){\hat{a}}_{Lo_H}(t')\big \rangle \right) , \end{aligned}$$

where $\big \langle {\hat{a}}_{Lo_H}^\dagger (t'){\hat{a}}_{Lo_H}(t')\big \rangle$ represents the photon-flux of the local oscillator, given by $\left| \alpha _{Lo}\right| ^2$. The photon-flux operator for the horizontal polarization state at fiber output for the quantum signal or pilot tone is given by

14 $$\begin{aligned} \big \langle {\hat{a}}_{B_H}^\dagger (t'){\hat{a}}_{B_H}(t')\big \rangle= & {} \frac{\tau _{ch}}{2}\left| {\mathbf {Z}}_{21}^{(n)}e^{i\frac{V_B^{(n)}}{V_\pi }\pi }+{\mathbf {Z}}_{11}^{(n)}\sqrt{\varepsilon _{\text {PDL}}}\right| ^2 \big \langle {\hat{a}}_{A_H}^\dagger (t'){\hat{a}}_{A_H}(t') \big \rangle \nonumber \\&\quad + \frac{\tau _{ch}}{2}\left| {\mathbf {Z}}_{22}^{(n)} e^{i\frac{V_B^{(n)}}{V_\pi }\pi }+{\mathbf {Z}}_{12}^{(n)}\sqrt{\varepsilon _{\text {PDL}}}\right| ^2\big \langle {\hat{a}}_{A_V}^\dagger (t'){\hat{a}}_{A_V}(t') \big \rangle \nonumber \\&\quad + \frac{\tau _{ch}}{2} \left( {\mathbf {Z}}^{*(n)}_{21}e^{-i\frac{V_B^{(n)}}{V_\pi }\pi }+ {\mathbf {Z}}^{*(n)}_{11}\sqrt{\varepsilon _{\text {PDL}}}\right) \left( {\mathbf {Z}}_{22}^{(n)}e^{i\frac{V_B^{(n)}}{V_\pi }\pi }+{\mathbf {Z}}_{12}^{(n)}\sqrt{\varepsilon _{\text {PDL}}}\right) \big \langle {\hat{a}}_{A_H}^\dagger (t'){\hat{a}}_{A_V}(t')\big \rangle \nonumber \\&\quad + \frac{\tau _{ch}}{2}\left( {\mathbf {Z}}^{*(n)}_{22}e^{-i\frac{V_B^{(n)}}{V_\pi }\pi } +{\mathbf {Z}}^{*(n)_{12}} \sqrt{\varepsilon _{\text {PDL}}}\right) \left( {\mathbf {Z}}_{21}^{(n)}e^{i\frac{V_B^{(n)}}{V_\pi }\pi }+{\mathbf {Z}}_{11}^{(n)}\sqrt{\varepsilon _{\text {PDL}}}\right) \big \langle {\hat{a}}_{A_V}^\dagger (t'){\hat{a}}_{A_H}(t')\big \rangle . \end{aligned}$$

On the other hand, the photon-flux operator representing the quantum signal or pilot tone for the vertical polarization state at fiber output can be written as,

15 $$\begin{aligned} \big \langle {\hat{a}}_{B_V}^\dagger (t'){\hat{a}}_{B_V}(t')\big \rangle= & {} \frac{\tau _{ch}}{2}\left| {\mathbf {Z}}_{21}^{(n)}e^{i\frac{V_B^{(n)}}{V_\pi }\pi }-{\mathbf {Z}}_{11}^{(n)}\sqrt{\varepsilon _{\text {PDL}}}\right| ^2 \big \langle {\hat{a}}_{A_H}^\dagger (t'){\hat{a}}_{A_H}(t') \big \rangle \nonumber \\&\quad + \frac{\tau _{ch}}{2}\left| {\mathbf {Z}}_{22}^{(n)} e^{i\frac{V_B^{(n)}}{V_\pi }\pi }-{\mathbf {Z}}_{12}^{(n)}\sqrt{\varepsilon _{\text {PDL}}}\right| ^2\big \langle {\hat{a}}_{A_V}^\dagger (t'){\hat{a}}_{A_V}(t') \big \rangle \nonumber \\&\quad + \frac{\tau _{ch}}{2} \left( {\mathbf {Z}}^{*(n)}_{21}e^{-i\frac{V_B^{(n)}}{V_\pi }\pi }- {\mathbf {Z}}^{*(n)}_{11}\sqrt{\varepsilon _{\text {PDL}}}\right) \left( {\mathbf {Z}}_{22}^{(n)}e^{i\frac{V_B^{(n)}}{V_\pi }\pi }-{\mathbf {Z}}_{12}^{(n)}\sqrt{\varepsilon _{\text {PDL}}}\right) \big \langle {\hat{a}}_{A_H}^\dagger (t'){\hat{a}}_{A_V}(t')\big \rangle \nonumber \\&\quad + \frac{\tau _{ch}}{2}\left( {\mathbf {Z}}^{*(n)}_{22}e^{-i\frac{V_B^{(n)}}{V_\pi }\pi }-{\mathbf {Z}}^{*(n)}_{12} \sqrt{\varepsilon _{\text {PDL}}}\right) \left( {\mathbf {Z}}_{21}^{(n)}e^{i\frac{V_B^{(n)}}{V_\pi }\pi }-{\mathbf {Z}}_{11}^{(n)}\sqrt{\varepsilon _{\text {PDL}}}\right) \big \langle {\hat{a}}_{A_V}^\dagger (t'){\hat{a}}_{A_H}(t')\big \rangle . \end{aligned}$$

In Eq. (14) and in Eq. (15), the photon-flux at Alice output is given by

16a $$\begin{aligned} \big \langle {\hat{a}}_{A_H}^\dagger (t'){\hat{a}}_{A_H}(t') \big \rangle&= \frac{1}{2}\eta _{\text {MZM}}^{(n)} \left| h(t'-nT_s)\right| ^2 |\alpha _s|^2 \end{aligned}$$

16b $$\begin{aligned} \big \langle {\hat{a}}_{A_V}^\dagger (t'){\hat{a}}_{A_V}(t') \big \rangle&= \frac{1}{2}\varepsilon _{\text {PDL}}\eta _{\text {MZM}}^{(n)} \left| h(t'-nT_s)\right| ^2 |\alpha _s|^2 \end{aligned}$$

16c $$\begin{aligned} \big \langle {\hat{a}}_{A_H}^\dagger (t'){\hat{a}}_{A_V}(t') \big \rangle&= \frac{1}{2}\sqrt{\varepsilon _{\text {PDL}}}\sqrt{\eta _{\text {MZM}}^{(n)}}e^{-i\frac{V_A^{(n)}}{V_\pi }\pi }\left| \alpha _s\right| ^2\left| h(t'-nT_s)\right| ^2 \end{aligned}$$

16d $$\begin{aligned} \big \langle {\hat{a}}_{A_V}^\dagger (t'){\hat{a}}_{A_H}(t') \big \rangle&= \frac{1}{2}\sqrt{\varepsilon _{\text {PDL}}}\sqrt{\eta _{\text {MZM}}^{(n)}}e^{+i\frac{V_A^{(n)}}{V_\pi }\pi }\left| \alpha _s\right| ^2\left| h(t'-nT_s)\right| ^2. \end{aligned}$$

In addition to the quadratures voltages defined in Eq. (11), we can also obtain the Stokes parameters for each transmitted symbol *n*. The Stokes parameters allow us to characterize the polarization state after homodyne detection at Bob detection system in Fig. 1. This is essential to assess the impact of the PMD on the pilot tone during its evolution over the transmission channel. Mapping the polarization state obtained for the pilot tone allows to implement appropriate polarization compensation techniques. The total intensity of the transmitted pilot-pulse *n* is defined by the Stokes parameter ${\hat{S}}_{0,n}$ which can be expressed as following^53^

17 $$\begin{aligned} {\hat{S}}_{0,n} = \left( {\hat{Q}}_{H,n}+ i {\hat{P}}_{H,n} \right) ^\dagger \left( {\hat{Q}}_{H,n}+i{\hat{P}}_{H,n}\right) + \left( {\hat{Q}}_{V,n}+i{\hat{P}}_{V,n}\right) ^\dagger \left( {\hat{Q}}_{V,n}+i{\hat{P}}_{V,n}\right) . \end{aligned}$$

The three-dimensional vector $\left( {\hat{S}}_{1,n},{\hat{S}}_{2,n},{\hat{S}}_{3,n}\right)$ divided by the total intensity of each transmitted pulse *n* (see Eq. 17) denotes the location of the state of polarization on Poincaré sphere with coordinates

18a $$\begin{aligned} {\hat{S}}_{1,n}&= \left( {\hat{Q}}_{H,n}+ i {\hat{P}}_{H,n} \right) ^\dagger \left( {\hat{Q}}_{H,n}+i{\hat{P}}_{H,n}\right) - \left( {\hat{Q}}_{V,n}+i{\hat{P}}_{V,n}\right) ^\dagger \left( {\hat{Q}}_{V,n}+i{\hat{P}}_{V,n}\right) , \end{aligned}$$

18b $$\begin{aligned} {\hat{S}}_{2,n}&= \left( {\hat{Q}}_{V,n}+ i {\hat{P}}_{V,n} \right) ^\dagger \left( {\hat{Q}}_{H,n}+i{\hat{P}}_{H,n}\right) + \left( {\hat{Q}}_{H,n}+i{\hat{P}}_{H,n}\right) ^\dagger \left( {\hat{Q}}_{V,n}+i{\hat{P}}_{V,n}\right) , \end{aligned}$$

18c $$\begin{aligned} {\hat{S}}_{3,n}&= -i\left( \left( {\hat{Q}}_{V,n}+ i {\hat{P}}_{V,n} \right) ^\dagger \left( {\hat{Q}}_{H,n}+i{\hat{P}}_{H,n}\right) - \left( {\hat{Q}}_{H,n}+i{\hat{P}}_{H,n}\right) ^\dagger \left( {\hat{Q}}_{V,n}+i{\hat{P}}_{V,n}\right) \right) . \end{aligned}$$

From the quantum state of polarization Stokes coordinates in Poincaré sphere, we can have information about the current location of the state without the need of additional signals, which allows us to have knowledge about the suffered drift though the quantum transmission channel. In this way, we can track the pilot signal and easily find the reversal polarization random drift operator and compensate it by performing a deterministic rotation on the EPC at the Bob’s input in Fig. 1.

**Figure 2:**
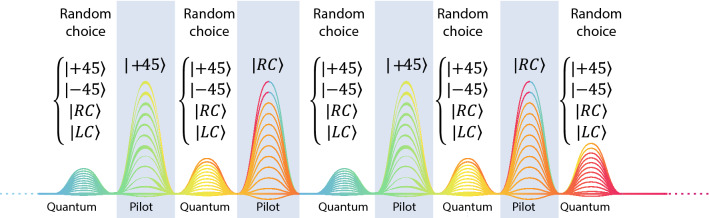
Transmitted frame where the pilot tone that follows a deterministic sequence alternating between $|45\rangle$ and $|RC\rangle$ is time-multiplexed with the quantum data signal, which the sequence is randomly chosen between four possible SOPs.

**Figure 3:**
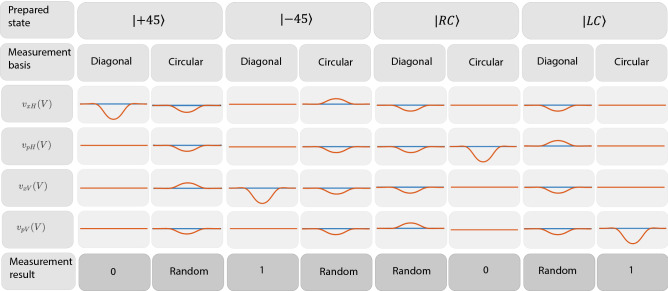
Representative schematics for the voltages at TIA’s output in Fig. 1 for each of the four prepared states considering one of the two measurement basis, and the corresponding bit measurement result.

## Discussion

### DV-QKD BB84 protocol implementation using homodyne detection

In this sub-section, we detail the DV-QKD BB84 protocol implementation. The DV-QKD BB84 is a prepared-measured protocol that requires the preparation of four states of polarization obtained from two non-orthogonal mutually unbiased bases. In this work, we consider the diagonal and circular bases. When Alice and Bob choose the same polarization basis, the homodyne detection output is deterministic. For instance, in the diagonal basis the $|45\rangle$ and the $|-45\rangle$ polarization states will be measured in Fig. 1 by the homodyne detectors $v_{x_H}^{(n)}(t)$ and $v_{x_V}^{(n)}(t)$, respectively. On the other hand, when Alice and Bob use the circular basis the $|RC\rangle$ and $|LC\rangle$ polarization states will be measured in Fig. 1 by the homodyne detectors $v_{p_H}^{(n)}(t)$ and $v_{p_V}^{(n)}(t)$, respectively. When Alice and Bob bases are not coincident, the measurement is random. Figure 3 summarizes the possible outcomes of the measurement results. Moreover, in terms of binary, the bit 0 is obtained whenever the $|+45\rangle$ or $|\text {RC}\rangle$ are prepared in Alice’s side, and the diagonal or circular measurement basis is chosen in Bob’s phase modulator, respectively. The bit 1 is obtained whenever the $|-45\rangle$ or $|\text {LC}\rangle$ are prepared in Alice’s side, and the diagonal or circular measurement basis is chosen in Bob’s phase modulator, respectively. Besides that, when the state of polarization in Alice’s side is prepared in a different basis than the selected measurement basis in Bob’s side, a random outcome is obtained. Since the preparation and measurement bases are orthogonal, the single-photon has a 1/2 probability of emerging in ${\hat{c}}_{H}$ and a 1/2 probability of emerging in ${\hat{c}}_V$ in Fig. 1.

The implemented protocol comprises two time-multiplexed signals, see Fig. 2. The pilot tone (classical optical signal) is implemented assuming $\eta _{\text {MZM}}=1$ in the MZM. The pilot tone is used to compensate the phase and frequency mismatches between Alice and Bob lasers, and also for characterize the polarization drift imposed by the optical fiber. The polarization drift compensation can be achieved assuming that for the pilot tone Alice and Bob agrees in a previously established sequence of polarization states, see for instance Fig. 2. In order to prepare this pilot tone, Alice alternatively applies $V_A=0$ V and $V_A=-V_{\pi /2}$ in its phase modulator to send $|+45\rangle$ and $|\text {RC}\rangle$ polarization states, respectively. Bob measures the pilot tone alternatively (not randomly) applying $V_B=0$ V and $V_B=V_{\pi /2}$ to choose the diagonal and circular basis, respectively. From the difference between what Bob measures and the ideal scenario without fiber PMD, Bob can use that information to reverse the fiber polarization drift using the EPC in Fig. 1. The pilot tone is time-multiplexed with the quantum signal in consecutive transmitted symbols.

The quantum signal is prepared choosing a very low efficiency in the Alice’s MZM amplitude modulator, which is calculated according with Eq. (2), such that at Alice output we have $\langle n_Q\rangle = 0.2$ photons per pulse. For the quantum signal implementation, Alice randomly chooses one of the four voltages for preparing one of the four considered states of polarization: $V_A=0$ V or $V_A=V_\pi$ V to prepare $|+45 \rangle$ or $|-45 \rangle$, respectively, and $V_A= V_{\pi /2}$ V or $V_A= -V_{\pi /2}$ V to prepare $|\text {RC} \rangle$ or $|\text {LC} \rangle$, respectively. For quantum pulses measurement, the measurement basis is also chosen in a random fashion. Bob randomly chooses between the diagonal basis, applying $V_B=0$ V, or the circular basis applying $V_B=V_{\pi /2}$ V.

### Polarization drift compensation

**Figure 4:**
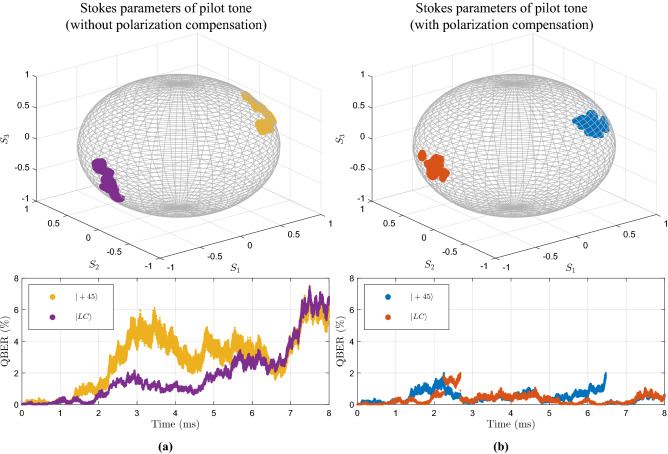
Poincaré sphere representation of the evolution of the SOPs $|45\rangle$ and $|\text {RL}\rangle$ sent in the pilot tone, and the respective QBER of each SOP over time. We consider that 8 million of symbols were transmitted, where the pilot tone is time multiplexed with the quantum signal. The polarization random drift was modelled using a $\sigma ^2=2\times 10^{-9}$ to obtain the matrix $M_F$ in Eq. (4) for each transmitted symbol. In this simulation we consider a 40 km standard optical fiber channel. **(a)** Represents the SOPs and QBER evolution for a simulation without an active compensation of the EPC at Bob’s input in Fig. 1. **(b)** Represents the SOPs and QBER evolution for a simulation with an active polarization compensation on the EPC in Fig. 1 using the Stokes parameters calculated with Eq. (18) for each transmitted pilot signal *n*.

Polarization mode dispersion is a serious obstacle on practical polarization encoded based communication system over optical fiber networks. In this work, we take advantage of continuous Stokes parameters information, measured from the obtained quadrature, and calculated according with Eq. (18), to reconstruct the received state of polarization and compensate the polarization random drift due to PMD. In order to find the polarization random drift reversal operator, we monitor the deterministic sequence sent in the pilot tone that contains two states of polarization from two non-orthogonal mutually unbiased bases and apply the needed compensation rotations to maintain the QBER bellow the defined error boundary due to polarization random drift. In this work, we consider a boundary of $2\%$ error above which a compensation rotation must be applied using for instance an EPC. The polarization random drift velocity is modelled considering a $\sigma ^2=2\times 10^{-9}$ to obtain the the matrix $M_F$ for each transmitted symbol *n*, which induces a continuous drift on the prepared states of polarization when travel over a 40 km standard single-mode optical fiber with an attenuation coefficient of 0.2 dB/km. That value for $\sigma ^2$ maintains the QBER bellow the defined boundary for a little more that 2 ms, which is a typically value for a buried fiber subjected to external perturbations. We at Alice side an optical power at of $P_s=3$ mW at laser output, a symbol duration of $T_s=1$ ns, and for the pilot tone we use $\eta _{\text {MZM}}=1$. Moreover, at Bob detection system we consider a detection efficient per homodyne detection of $76 \%$, a TIA gain of $g_{\text {TIA}}=16^3$ V/A and bandwidth of $B_e=1.6$ GHz. besides that, for each transmitted symbol (pilot tone or quantum signal) we generated the electronic noise contribution from a Gaussian distribution with variance $\sigma ^2_{{\hat{Q}}_{e,n}}=0.4\times 10^{-3}V^2$ and zero mean^51^. The shot-noise contribution is independently simulated for each homodyne detector for each transmitted symbol from a Gaussian distribution with zero mean and variance calculated according with the variance of the four quadratures as presented in Eq. (12). We consider a PDL value of $\varepsilon _{\text {PDL}}=2.3$ dB. Figure 4 shows the stokes parameters obtained for the pilot tone states of polarization with and without active polarization compensation on the EPC at Bob’s input. Moreover, the QBER for each transmitted symbol *n* can be calculated from the stokes parameters obtained in relation to a reference state of polarization according with the following^54^,

19 $$\begin{aligned} \text {QBER}(\theta ,\phi ) = 1- \frac{1}{2}\Big ( 1+ \cos {\theta } \cos {\phi } \Big ), \end{aligned}$$

where $\theta = \arctan {\frac{\hat{S_2}}{\hat{S_1}}}$ and $\phi =\arcsin {\hat{S_3}}$. In the top of Fig. 4a, the pilot tone Stokes parameters without polarization drift compensation is shown, which correspond to a temporal evolution of QBER represented in the bottom of Fig. 4a. On the other hand, in the top of Fig. 4b, the pilot tone Stokes parameters considering an active compensation using the EPC at Bob side is shown. In the bottom of Fig. 4b the corresponding QBER is presented. The implemented polarization random drift compensation method guarantees a QBER bellow the defined error boundary due PMD for the total acquisition time. The method for polarization drift compensation presented in this work is free of additional hardware or extra bandwidth signals, since it uses the pilot tone states of polarization, which is already needed for phase and amplitude differences compensation between the transmitter laser and the locally generated local oscillator.

### Conjugate homodyne detection in counting mode

**Figure 5:**
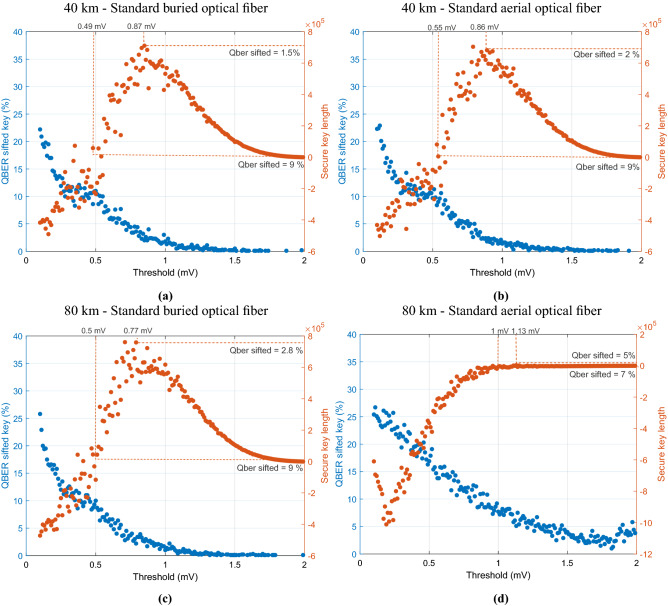
QBER of the sifted key and secure key length as a function of the voltage threshold applied in the quadratures calculated using Eq. (11). We consider that 8 million of symbols were transmitted, where the pilot tone is time multiplexed with the quantum signal. For the pilot tone we assume $P_s=3$ mW with $\eta _{\text {MZM}}=1$, whereas for the quantum signal we use $P_s=3$ mW and $\eta _{\text {MZM}}=9.83\times 10^{-5}$. In (**a**) and (**c**), it was consider a $\sigma ^2=2\times 10^{-9}$ to obtain the matrix $M_F$ in Eq. (4) for each transmitted symbol, which corresponds to a standard burried optical fiber. In (**b**) and (**d**), it was consider a $\sigma ^2=6\times 10^{-9}$ to obtain the matrix $M_F$ in Eq. (4) for each transmitted symbol, which corresponds typically to an aerial optical fiber implementation.

The DV-QKD protocols demand to discriminate the vacuum state from non-vacuum states. In order to operate the conjugate homodyne detection scheme in photon counting mode, the continuous detection measurements must be mapped to one of the two possible events, click or no-click. In this work, we adopt a strategy based on pre-defined detection threshold, $\tau \in \{0,\infty \}$, above which we consider a click and below which we consider no-click. That mapping process is software implemented in the post-processing stage. By choosing the appropriate $\tau$ we aim the longer secure key with a lower sifted key QBER in the DV-QKD BB84 protocol.

The basic idea of BB84 protocol is the exchange of two set of states orthogonal within each set with a 1/2 probability of overlap between sets. Since the receiver randomly chooses the measurement basis, Bob and Alice obtain a raw key that after being distilled results in a sifted key after publicly perform basis reconciliation. Figure 5 shows the QBER calculated using 1000 bits from the sifted key, which are later discarded before obtain the final secure key, as a function of the chosen threshold $\tau$. Please note that for the quantum signal we are using $\eta _{\text {MZM}}=9.83\times 10^{-5}$ which allows us to obtain $\langle n_Q \rangle$=0.2 photons per pulse for the quantum signal. The system was simulated considering two different distances for the quantum channels, 40 km and 80 km, assessing a buried optical fiber and a standard aerial optical fiber for each distance. In this way, the curves of the QBER of the sifted key and the secure key length as a function of the defined voltage threshold was obtained in Fig. 5a,c considering a standard buried optical fiber channel ($\sigma ^2=2\times 10^{-9}$), and in Fig. 5b,d considering an aerial optical fiber subject to heavy external conditions ($\sigma ^2=6\times 10^{-9}$). In this work, we consider the power of eavesdropper is limited to an individual attack for realistic signal sources^9^, where Eve uses the single-photon detectors operating in gated mode commonly used in standard DV-QKD implementations. In this way, we consider that the error correction code has a practical efficiency of $f_{EC}=1.2$, and the estimated portion of the sifted key disclosed is $\text {leak}_{EC}=f_{EC}h(E)$, where *h*(*E*) is the binary Shannon entropy of the observed error rate *E*. Moreover, we also consider that the estimated error rate from a sifted key of size *N* may be deviated from the actual value with probability $\varepsilon _{PE}$ and can be given as $\tilde{E}=E+\frac{1}{2}\sqrt{\{2\ln {(1/\varepsilon _{PE})}+2\ln {(N+1)}\}(1/N)}$. The secure key length in Fig. 5 is calculated as following^55^

20 $$\begin{aligned} l = N \Big (1-h\big (\tilde{E}\big ) \Big ) - N \text {leak}_{EC}-7N\sqrt{\frac{1}{N}\log _2\frac{2}{\tilde{\varepsilon }}}-2\log _2\frac{1}{\varepsilon _{PA}}-\log _2\frac{2}{\varepsilon _{EC}}, \end{aligned}$$

where $\varepsilon =\varepsilon _{PE}+\tilde{\varepsilon }+\varepsilon _{PA}+\varepsilon _{EC}$ is a security parameter, $\tilde{\varepsilon }$ is the probability that information of Eve is underestimated when using smooth min-entropy, $\varepsilon _{PA}$ is the collision probability of two different input strings can be projected into the same outcome, and $\varepsilon _{EC}$ is the probability failure of the error correction code.

As one can see in all Fig. 5a–d there is an optimum threshold value $\tau$ that leads to the longer secure key obtained with the presented DV-QKD system that does not correspond to the minimum sifted key QBER. It is certain that increasing the threshold leads to less errors on the raw key and consequently on the sifted key. However, a high value for $\tau$ leads to a decrease on the secret key length. In Fig. 5a,b a positive secure key length is obtained for a QBER lower than  $9\%$. In this way, the minimum threshold applied to obtain a valid secure key length should be higher than 0.49 mV, which sets the zero secure key length. Moreover, a maximum on the secure key length for a 40 km optical fiber channel is achieved for a QBER of approximately of $1.5\%$. This corresponds to a detection threshold of approximately 0.87 mV. In addition, the robustness of the presented system is clear when one compares Fig. 5a with Fig. 5b. The proposed polarization drift compensation algorithm allows the large deployment of the presented scheme even considering heavy external perturbation that lead to a fast polarization drift, without consuming more bandwidth neither to use extra hardware. Finally, we can also see from Fig. 5a that for a system operating at 500 MHz symbol generation clock (considering pilot tone and quantum signal), a secure key length of 750 kbits was generated over approximately 16 ms, with a sifted $1.5\%$ sifted QBER, and a detection threshold of 0.87 mV. Considering a longer optical fiber channel, for a 80 km buried optical fiber channel, a maximum secure key length of 698 kbits is generated over approximately 16 ms with a QBER of  $2.8\%$, and applying a voltage threshold of 0.77 mV. Moreover, even considering heavy external perturbations the proposed system is able to generate a secure key with a maximum length of 4.3 kbits over 8 ms with a QBER of $5\%$ applying a threshold of 1.13 mV. Moreover, when we increase the quantum optical fiber channel length assuming a standard buried optical fiber channel the system shows a decrease of approximately $7\%$ on the final secret key length. However, for heavy external environments, the system is more sensible to the increase of the length of the quantum optical fiber channel, see Fig. 5c,d.

## Conclusion

In this paper, we present a novel polarization based DV-QKD system that combines the implementation of quantum states of polarization using phase-modulators with a polarization diversity coherent detection scheme. The deployment of weak quantum signals at high baud-rate are obtained with commercial Mach–Zehnder amplitude modulators followed by a $45^{\circ }$ aligned phase-modulator allowing to switch between states of polarization. On the receiver side the switching of the basis measurement is also performed by a commercial phase-modulator and the states of polarization are measured using standard homodyne detectors. In this way, the proposed system exclusively requires classical hardware, which allows its large deployment in current practical optical fiber networks. Besides the proposed system uses polarization encoding single-photons, the proposed system can also be used for time-bin encoding systems^56–58^.

In order to implement the BB84 protocol in the proposed system, two sets of states of polarization orthogonal within each set, and from two non-orthogonal mutually unbiased basis between sets are prepared and measured. Furthermore, the proposed scheme also implements a quantum frame where a time-multiplexed pilot signal is transmitted for phase and amplitude difference compensation between parties, and also for polarization random drift compensation. We showed that the implemented polarization compensation algorithm provides robustness to the implemented system without demanding extra bandwidth consumption, since it is quite insensible to heavy external perturbation. That feature stems from the capability of continuously locate the received SOP though the precise calculation of the Stokes parameters. We implement the DV-QKD BB84 protocol considering 1 GHz clock SOP generation, coherent state source heavily attenuated, electronic and shot noise contributions on the detection scheme, and error correction efficiency different from the Shannon limit. Considering the results in this work, we showed that for a system operating at 500 MHz symbol generation clock (considering pilot tone and quantum signal), a secure key length of 750 kbits was generated over approximately 16 ms, with a $1.5\%$ sifted QBER, and a detection threshold of 0.87 mV. In this way, the proposed system is able to generate secure keys at a rate of 46.9 Mbps with a QBER on the sifted key of $1.5\%$.
